# Bioluminescence may be affected by strain assembly history in a marine symbiont

**DOI:** 10.1093/ismeco/ycag279

**Published:** 2026-10-01

**Authors:** Emma D Román, Andrea N Nebhut, Tadashi Fukami, Alison L Gould

**Affiliations:** Department of Biology, Temple University, Philadelphia, PA 19122, United States; Department of Biology, Stanford University, Stanford, CA 94395, United States; Institute for Biodiversity Science and Sustainability, California Academy of Sciences, San Francisco, CA 94118, United States; Department of Biology, Stanford University, Stanford, CA 94395, United States; Division of Biosphere Sciences & Engineering, Carnegie Science, Pasadena, CA 91125, United States; Department of Biology, Stanford University, Stanford, CA 94395, United States; Department of Biology, Temple University, Philadelphia, PA 19122, United States; Institute for Biodiversity Science and Sustainability, California Academy of Sciences, San Francisco, CA 94118, United States

**Keywords:** priority effects, strain-level diversity, bacterial strain ecology, historical contingency, intraspecific interactions, microbial symbiosis, bioluminescence, mutualism, *Photobacterium mandapamensis*, *Siphamia tubifer*

## Abstract

Symbionts can be highly variable in their species or strain composition in ways that environmental factors alone cannot explain. One potential mechanism that contributes to this variability is priority effects, whereby the arrival order and timing influence how species or strains affect one another in the host. Here, we investigated whether priority effects influence strain composition in the luminous bacterial symbiont *Photobacterium mandapamensis*, which inhabits the light organ of the cardinalfish *Siphamia tubifer*. Using three strains isolated from the same individual host, we conducted *in vitro* experiments manipulating arrival order and initial abundance. Strains introduced earlier or at higher initial abundance consistently dominated the resultant bacterial populations, indicating strong priority effects. The strains differed measurably in growth and light production. Moreover, the bioluminescence of mixed-strain populations was not predictable from the performance of strains grown individually and, in some cases, exceeded monoculture luminosity by up to 4000%. These results suggest that assembly history could create otherwise inexplicable variability in the strain composition of symbionts and their bioluminescent output, calling for *in vivo* investigations to study priority effects in the symbiosis.

## Introduction

In some microbial populations and communities, early-arriving taxa can gain an advantage by occupying available niches or modifying the local environment in ways that facilitate or inhibit the growth of later-arriving taxa [1]. This process, known as priority effects, can influence species and strain composition [1]. In host-associated microbial symbionts, the host environment represents the habitat in which microbes establish, and recent studies suggest that priority effects can shape the strain and species composition of these symbionts [2–12]. Even in a single-species population, strain composition can be affected by intraspecific priority effects [13, 14]. Since conspecific strains can differ greatly in such traits as metabolic capacity, competitive strategies, and host specificity [15–18], intraspecific priority effects can have functional consequences not just in multi-species communities, but also in multi-strain assemblages consisting of a single species.

A tractable system for studying priority effects on strain composition comes from the symbiosis between the cardinalfish *Siphamia tubifer* and its bacterial symbiont *Photobacterium mandapamensis*. The host, *S. tubifer*, is an Indo-Pacific cardinalfish that lives among the spines of sea urchins during the day. It has a specialized, gut-associated light organ that houses the luminous bacterium *P. mandapamensis* [19, 20]*.* The bacterially produced light is used when the fish leaves the urchin at night, presumably to match downwelling moonlight for camouflage [19, 20]. Larvae acquire symbionts from the environment during their pelagic phase after ~1 week of development, and *P. mandapamensis* is the only bacterium known to establish in the light organ of *S. tubifer* [19, 21, 22]. Despite the specificity of this binary relationship, individual fish host diverse strain assemblages within their light organ. Each light organ contains an average of five to six *P. mandapamensis* strains, with minimal strains shared between individual hosts [22]. Given that symbionts are environmentally acquired, the timing and order in which the host encounters strains may determine which genotypes successfully establish in the light organ. Furthermore, because multiple closely related strains coexist within individual hosts, this system offers a tractable model for testing how interactions among conspecific strains may shape intraspecific composition and functional output.

Here, we investigate how priority effects influence the assembly and bioluminescent function of *P. mandapamensis* strains. Using three strains isolated from a single *S. tubifer* light organ [22], we conducted *in vitro* experiments manipulating arrival order and initial abundance as an initial step toward understanding priority effects in this symbiotic bacterium. The *in vitro* approach had logistical advantages, enabling control over initial abundance and arrival timing, high temporal resolution, and direct measurement of luminescence, which are difficult to achieve *in vivo.* We first quantified growth rate and bioluminescence in monocultures to determine whether strains differ in ecologically relevant phenotypes. We then examined how these strains interact in mixed cultures to determine if assembly history influences light production. We hypothesized that differences in colonization history would alter strain composition and lead to measurable differences in bioluminescent output.

## Materials and methods

### Bacterial strains and fluorescent tagging

We tested assembly processes and bioluminescent function in three *P. mandapamensis* strains, StP 4.11 (Ph.E), StP 4.7 (Ph.O), and StP 4.16 (Ph.P), isolated from the light organ of the same *S. tubifer* individual collected from Verde Island in the Philippines [22]. These strains were defined using ERIC-PCR and further resolved using whole-genome sequencing in a previous study, which revealed that the strains are genetically similar to one another with an average nucleotide identity greater than 97% [22]. Nonetheless, the strains differed in relative abundance within the host light organ, with Ph.E accounting for 81%, Ph.O 5%, and Ph.P 14% [22], allowing us to examine differences in assembly and bioluminescent function among closely related symbionts. To enable tracking in co-culture experiments, strains Ph.E and Ph.O were engineered to constitutively express the fluorescent proteins mVenus and mRuby, respectively, by the San Diego State University’s Research Foundation [23, 24]. Growth rates of the engineered strains were unaffected by the fluorescent tag (Supplementary Fig. S1).

### Strain phenotype assays

#### Growth rate

We measured growth rates using a BioTek Synergy H1 Hybrid Multi-Mode Microplate Reader and BioTek Gen5 Microplate Reader and Imager software (v3.10). Wild-type strains were revived from −80°C glycerol stocks, streaked onto agar plates containing lysogeny broth, Lennox formulation, prepared in 70% seawater (LSW-70), and incubated at 26°C for 24 h. Single colonies of each strain were inoculated into 3 ml of LSW-70 liquid media and grown overnight. Cultures were then standardized to an OD_600_ of 0.7, diluted 1:100, and added to a 96-well plate. Uninoculated media (100 μl) were used as blanks. Absorbance readings at 600 nm were then taken every 5 min for 24 h at room temperature with the lid on to reduce evaporation. Growth curves of the wild-type strains were analyzed in R (v4.2.1) using the *growthcurver* package [25], and we compared growth rates using an analysis of variance (ANOVA) with Tukey-adjusted post hoc comparisons of estimated marginal means. Estimated doubling times were calculated and are reported in hours. Growth rates represent the growth during exponential phase per hour and are reported in h^−1^.

#### Luminosity of strains

We quantified the luminosity of the strains individually and across multiple mixtures. Prior to each experiment, we grew the wild-type version of each strain overnight on LSW-70 agar plates at 26°C. The following morning, individual colonies were isolated and grown overnight in LSW-70 liquid media at 26°C. A total of eight isolates were used for each strain to compare individual strain performances. Of the eight isolates, two from each strain were randomly selected to be grown in 25 ml of liquid media. These isolates were used for both individual and mixed culture measurements. The remaining six isolates used solely for individual measurements were grown in only 3 ml of media. After 15 h, cultures were standardized using a Thermo Fisher Varioskan ALF Multimode Microplate Reader with accompanying SkanIt software. Next, we created mixed compositions by adding each strain at either a high (1000 μl) or low (10 μl) initial density to mirror the conditions used in the competition assays. All possible combinations, including the naturally occurring strain composition (81% Ph.E, 5% Ph.O, and 14% Ph.P) [22], were created.

After the preparation of wild-type single- and multi-strain cultures, luminescence was immediately measured using a GloMax 20/20 Luminometer (Promega). For each sample, we agitated 1 ml aliquots for 5 s before each reading. All measurements were standardized to a blank which contained 1 ml of LSW-70 broth, and luminosity was reported in relative light units (RLUs). We analyzed luminosity measurements in R (v4.2.1) using an ANOVA and post hoc estimated marginal means with Tukey Honest Significant Difference (HSD) correction to compare strain assemblages.

### Sample preparation for assembly experiments

Overnight cultures of the fluorescently labeled Ph.E, Ph.O, and wild-type Ph.P strains were grown as previously described and standardized to an OD_600_ of 0.7. To investigate priority effects, we conducted two types of experiments: one manipulating the timing of strain introduction and another varying initial inoculum concentrations. These parallel approaches allowed us to evaluate whether differences in starting concentration can serve as a reliable proxy for arrival order in this system as demonstrated in others [26, 27]. To simulate differential arrival times, we used high (~10^4^ colony-forming units per milliliter [CFU/mL]) and low (~10^2^ CFU/ml) initial concentrations prepared through serial 1:10 dilutions, simulating early and late arrivals, respectively. In timing experiments, we introduced strains either simultaneously or with a 4-h delay to create staggered arrival scenarios. For these, we used the high concentration (~10^4^ CFU/ml) from the altered starting concentration experiments to optimize the efficiency of flow cytometry data collection. The 4-h delay was also chosen to coincide with the first flow cytometry measurements.

#### Pairwise assembly experiments

Prior to multi-strain experiments, we evaluated direct competition between strains in a series of pairwise experiments. For each pairing, strains were started at either a high (~10^4^ CFU/ml) or low (~10^2^ CFU/ml) initial concentration to assess the simulated effects of arrival order on final abundance. Experiments were conducted in clear, flat-bottom 96-well plates with 80 μl of sterile LSW-70 media added into each well. Pairwise combinations were created by adding 10 μl of each strain into the 96-well plate (Supplementary Table S1). As controls, we included wells containing only one strain at a 1:10 dilution or blank wells with 100 μl of sterile LSW-70 media. Absorbance was measured every 5 min for 24 h using a BioTek Gen5 Microplate Reader. To reduce evaporation, the lid of the 96-well plate remained on throughout the experiment. At 0, 10, and 24 h, we destructively sampled wells in triplicate from the 96-well plate, diluting 1:10 at 0 h and 1:10^6^ at 10 and 24 h. The diluted samples were plated on LSW-70 agar and incubated at 26°C for 24 h.

#### Multi-strain assembly experiments

To investigate intraspecific priority effects with all three strains present, we tested combinations of high and low starting densities in clear, flat-bottom 96-well plates (Supplementary Table S2). Wells received 70 μl of sterile LSW-70 media and 10 μl of high (~10^4^ CFU/ml) or low (~10^2^ CFU/ml) inoculum for each strain, depending on the desired combination. As controls, wells containing only one strain or blank wells with 100 μl of sterile LSW-70 media were included. Absorbance of the inoculated 96-well plates was measured with a BioTek Gen5 Microplate Reader at room temperature over 24 h. To reduce evaporation, the lid of the 96-well plate was kept on throughout the experiment. At 0, 10, and 24 h, we destructively sampled wells in triplicate, following the same dilution and plating protocols as described above.

To quantify strain abundance from the pairwise and three-way experiments, we enumerated colonies with a Dino-Lite fluorescence handheld microscope. Strains with the low starting concentration produced too few colonies to calculate CFU/ml due to plating limitations. When zero colonies were counted, we reassigned the counts to a value of 1 to reflect very low abundance rather than true absence.

### Flow cytometry assembly experiments

To enable a more nuanced analysis, we used flow cytometry to improve strain-level tracking and simulated priority effects by altering strain concentrations and staggering arrival orders. Experimental setups followed the mixed culture protocol, using fluorescently labeled Ph.E and Ph.O strains (Supplementary Tables S3 and S4). We used two 96-well plates to allow for three replicates per time point and grew both 96-well plates in a New Brunswick Innova 43/43 R Console Incubator Shaker at 120 rpm and 26°C. Optical density readings were taken every hour for 12 h and once at 24 h using a BioTek Synergy H1 Hybrid Multi-Mode Microplate Reader. Samples at 4, 6, 8, 10, 12, and 24 h were analyzed using an Attune NxT Flow Cytometer. We optimized the instrument settings for our mRuby and mVenus fluorescent tags and applied gating strategies to isolate single-cell events. To ensure minimal background fluorescence, we also analyzed blank wells containing sterile LSW-70 media and control wells containing each strain grown separately. Before analysis, samples were diluted 1:10 in phosphate-buffered saline (PBS), except for 4-h samples which ran undiluted. To minimize contamination, a 10% bleach solution was run between all samples. Each sample was passed through the flow cytometer at a rate of 12.5 μl/s until 100 000 events were collected. Afterwards, we recorded the abundance of non-fluorescent (Ph.P), mRuby (Ph.O), and mVenus (Ph.E) populations.

Flow cytometry data was prepared using the FlowJo software (v10.10, BD Biosciences). We ensured all experiments received the same gating parameters and exported the counts for each strain for further analysis. Bacterial abundance (counts/ml) was calculated using the same approach as the CFU/ml determination.

To assess whether differences in initial abundance or arrival order influenced strain composition, bacterial counts over time were analyzed using linear models in R (v4.2.1). Furthermore, to be consistent with the plating data presented in the Supplementary Information, we analyzed the relative abundance of each strain within each assemblage at the 10-h timepoint. We also chose the 10-h data to minimize the influence of dead cells, which we found to be more prevalent at later timepoints. Technical replicates were averaged and then the log-transformed counts/ml were assessed using linear models that included strain identity and a three-way interaction among the initial concentrations (or arrival order) of Ph.E, Ph.O, and Ph.P. Differences in relative abundance were evaluated using ANOVA, followed by post hoc pairwise comparisons of estimated marginal means with Tukey’s HSD correction.

### Luminosity over time

To complement static luminosity measurements, luminescence and OD_600_ were measured simultaneously over 24 h. Wild-type strains were streaked onto agar plates and grown overnight at 26°C. The following day, eight colonies from each plate were isolated, suspended in 3 ml of LSW-70 broth, and grown overnight. On the day of the experiment, all overnight cultures were standardized, diluted 1:100, and added to a clear-bottom, white 96-well plate. Strains were grown individually and in mixed cultures by introducing strains either initially or after 4 h. We recreated the naturally occurring strain composition described in Gould *et al*. [22], using 81% Ph.E, 5% Ph.O, and 14% Ph.P, and compared it with the experimental assemblages. The “original” strain composition was assembled at these proportions and introduced at the start of the experiment. The experimental assemblage that most closely approximated this composition under sequential addition was Early Ph.E – Late Ph.O – Late Ph.P. After the 96-well plate was inoculated with either single strains or the early arrival strains, the plate was sealed using an optically clear heat seal to minimize evaporation during the experiment. We used 100 μl of uninoculated media as blanks. Readings at 600 nm followed by luminescence readings were then taken every 15 min for 24 h at room temperature using a Thermo Fisher Varioskan ALF Multimode Microplate Reader with accompanying SkanIt software. After 4 h, we carefully removed the seal from the plate to spike the plate with the second arriving strains. All strains added at the 4-h mark were standardized and diluted the same as initial strains. We then sealed the plates with a new heat seal and continued the run.

Blanks were subtracted from the absorbance and luminosity measurements, and luminosity was standardized by absorbance (RLU/OD_600_) and plotted over time in R (v4.2.1). OD_600_ was also plotted over time to visualize bacterial growth dynamics across treatments. The percent change in luminescence was calculated relative to monoculture controls at each time point. For each observation, luminescence of a mixed strain assemblage was compared to the mean luminescence of the corresponding monoculture strain measured at the same time point using the formula: 


$$ \% change=\frac{\Big(\frac{RLU}{OD_{Mixture}}-\frac{RLU}{OD_{Reference\ Strain}}\Big)}{\frac{RLU}{OD_{Reference\ Strain}}}\times 100 \% $$

Positive values indicate higher luminescence than the monoculture reference, while negative values indicate reduced luminescence. The stability of light output was calculated as the coefficient of variation between 5 and 20 h for all mixtures. We analyzed all luminosity measurements in R (v4.2.1) using an ANOVA and post hoc estimated marginal means with Sidak correction to compare the light output and stability of mixed-strain groupings.

## Results

### Strains differed in growth rate and luminosity

The strains exhibited distinct phenotypes and differed significantly in exponential growth rate (h^−1^) (Fig. 1A). Ph.E had the highest average rate (0.83 h^−1^), corresponding to a doubling time of ~50 min. It grew significantly faster than both Ph.O (0.47 h^−1^; doubling time ~ 1.5 h, *P* < .001) and Ph.P (0.52 h^−1^; doubling time ~ 1.3 h, *P* < .001). Ph.O and Ph.P did not differ significantly in growth (*P* = .59). Although Ph.E was the fastest-growing strain overall, it also exhibited the widest variation, ranging from 0.59 to 0.99 h^−1^ (doubling times of ~1.2 h to ~42 min). The slowest growth rate observed across all strains was 0.40 h^−1^ in Ph.O (doubling time ~ 1.7 h).

**Figure 1:**
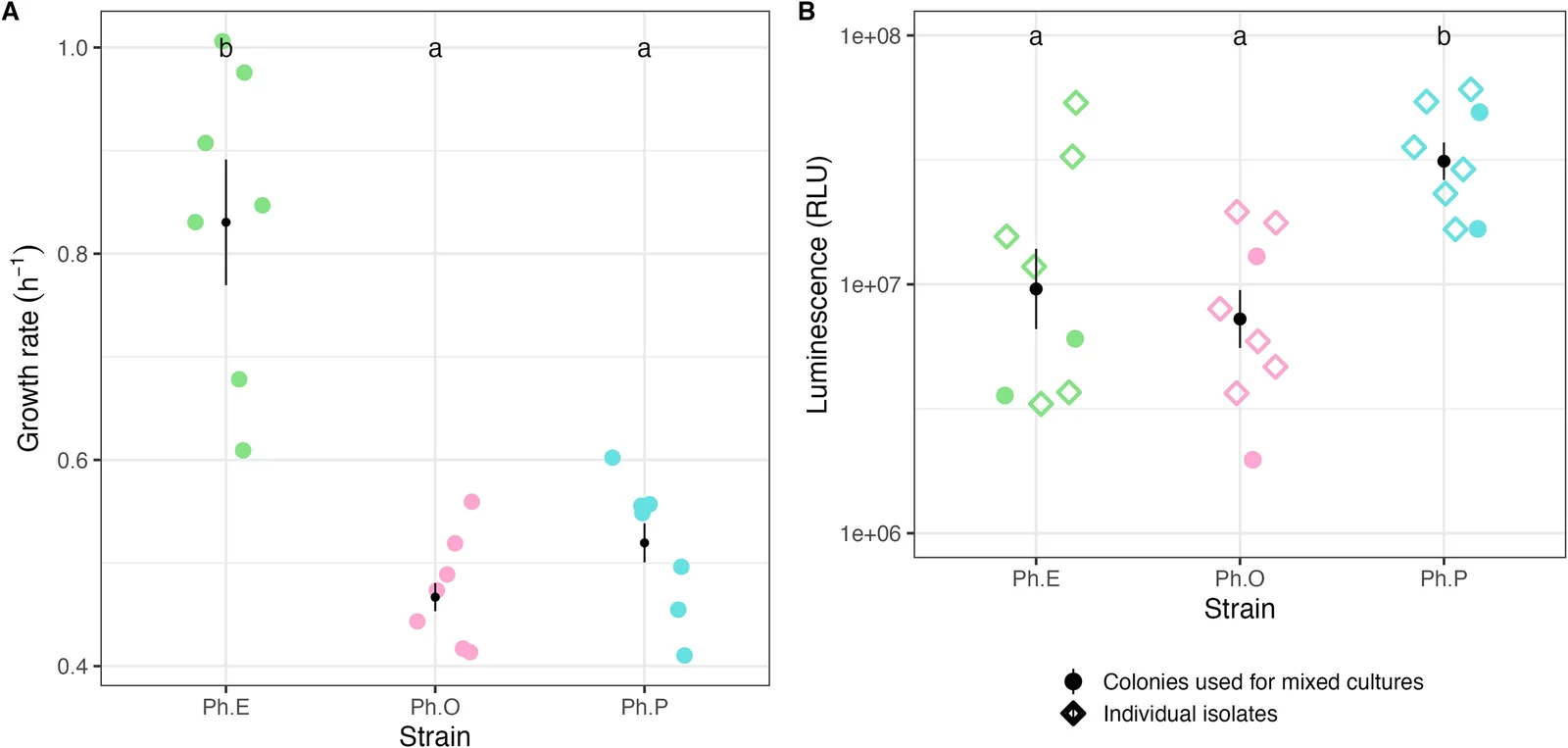
Growth rate (per hour, h^−1^, (A) and luminescence (relative luminosity units, RLU, (B) differed among wild-type *P. mandapamensis* strains grown individually; growth rates were estimated from 24-h growth curves (*n* = 7), and luminosity was measured from standardized overnight cultures (*n* = 8); in panel B, dots represent individual colonies used to assemble mixed cultures (Supplementary Fig. S5), while diamonds represent colonies measured only in isolation. Individual data points are shown with means ± standard error indicated by black bars; strains sharing the same letter are not significantly different from one another (*P* > .05).

Luminosity (RLU) also varied significantly among strains (Fig. 1B). Ph.P had an average luminosity of 3.4 × 10^7^ ± 5.8 × 10^6^ RLUs and was brighter than Ph.E (*P* = .02) and Ph.O (*P* = .004). Ph.E and Ph.O did not significantly differ in brightness (*P* = .77), averaging 1.6 × 10^7^ ± 5.8 × 10^6^ RLUs and 9.1 × 10^6^ ± 2.1 × 10^6^ RLUs, respectively. Similar to growth, Ph.E again exhibited the greatest variability, ranging from 3.0 × 10^6^ RLUs to 4.9 × 10^7^ RLUs. The lowest luminescence recorded was 2.0 × 10^6^ RLUs in Ph.O.

### Priority effects influenced strain composition

After growing in mixed cultures, the final strain composition closely reflected initial conditions (Fig. 2; Supplementary Figs S2–S4). In both pairwise and three-strain assemblages, priority effects shaped the final composition (Supplementary Figs S2 and S3). After 10 h, the strains introduced at a high initial concentration were consistently the most abundant, and this pattern held in pairwise competition, indicating that priority effects persist even in simple strain interactions (Supplementary Figs S2 and S3). While strains introduced at low concentration remained detectable, too few colonies were counted to reliably estimate CFU/ml (Supplementary Figs S2 and S3).

**Figure 2:**
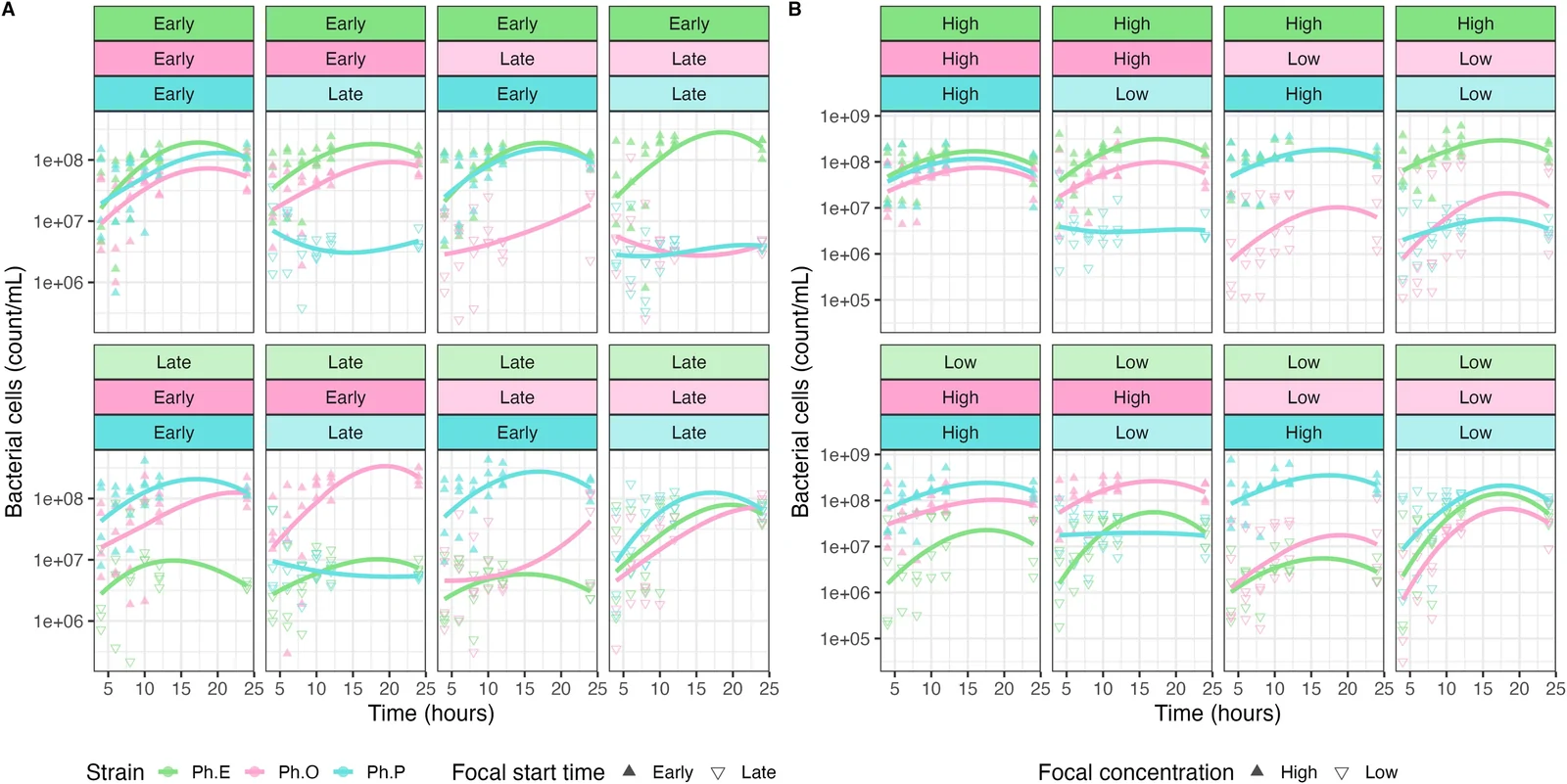
Initial abundance and arrival order influenced the composition of mixed *P. mandapamensis* strain cultures over 24 h; abundance was measured using flow cytometry; priority effects were simulated by staggering arrival order (A) or altering initial strain concentrations as a proxy for arrival order (B); the treatment conditions are indicated above each panel; upward-pointing triangles denote strains that arrived first or were introduced at a higher concentration; downward-pointing triangles indicate strains that arrived 4 h later or at a lower concentration; each point represents the average abundance across biological replicates (*n* = 3 for the all-late treatment at 10 and 12 h, *n* = 4 for all other treatments and timepoints), with a loess-smoothed best-fit curve with a span parameter of 1.5.

When measuring cell counts using flow cytometry, priority effects remained strong, and strain composition remained relatively constant over time, regardless of whether strain arrival order or initial concentration was manipulated (Fig. 2). Although one biological replicate was excluded from the 10- and 12-h timepoints of the altered timing experiment due to a technical error resulting in a missing signal, overall patterns remained consistent. Here, we highlight the 10-h results as they provide a clearer snapshot of strain composition rather than later time points which had more noise due to increased cell death. However, strain composition after 24 h showed consistent trends (Supplementary Fig. S4). Across treatments, no single strain consistently outcompeted the others, and there were no qualitative differences between experiments manipulating arrival order versus initial concentration (Fig. 3).

**Figure 3:**
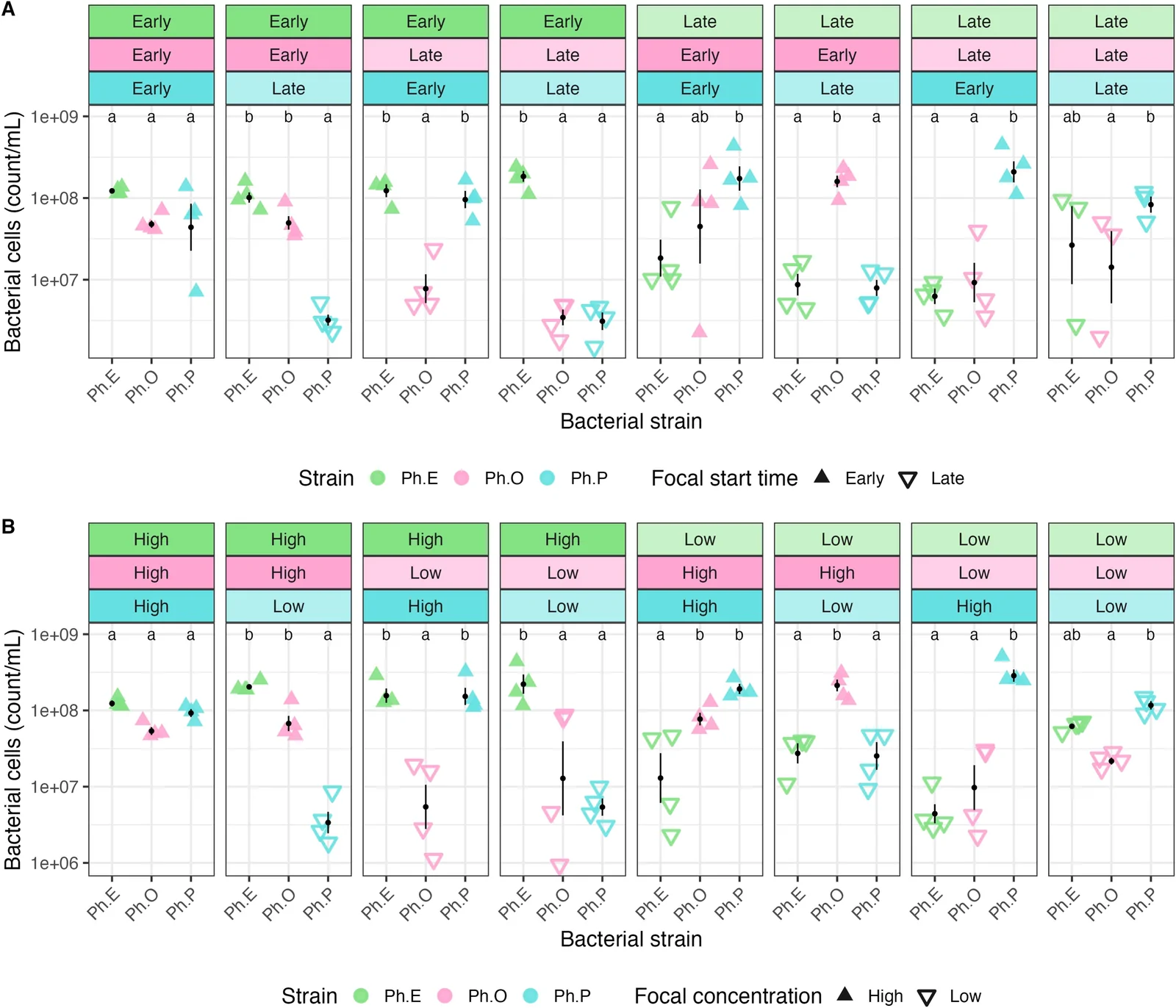
Initial abundance and arrival order influenced the composition of mixed *P. mandapamensis* strain cultures after 10 h of growth; strain abundance was measured using flow cytometry; priority effects were simulated by staggering arrival order (A) or altering initial strain concentrations as a proxy for arrival order (B); the treatment conditions are indicated above each panel; upward-pointing triangles denote strains that arrived first or were introduced at a higher concentration; downward-pointing triangles indicate strains that arrived 4 h later or at a lower concentration; each point represents the average abundance across biological replicates (*n* = 4, *n* = 3 for the all-late treatment), with black points representing means ± standard error indicated by black bars; strains sharing the same letter are not significantly different from one another (*P* > .05).

Ph.O exhibited the greatest variability in abundance across treatments (Fig. 3). For instance, Ph.O was less abundant than Ph.P when all strains were introduced late (*P* = .04) or at low concentrations (*P* = .01). These differences were no longer apparent at the 24-h time point (Supplementary Fig. S4). Additionally, Ph.O was not significantly more abundant than Ph.E when Ph.E was introduced late. However, under the equivalent conditions when concentration was manipulated, Ph.O was significantly more abundant than Ph.E (*P* = .01). This trend was also reflected in the 24-h data (Supplementary Fig. S4). Ph.P exhibited the most variability when all strains were introduced early but remained highly consistent across all other treatments.

### Strain mixing instantly influenced luminosity

Across mixtures with different strain compositions, luminescence varied when measured immediately after mixing (Supplementary Fig. S5, Supplementary Table S9). We recreated the naturally occurring strain composition [22], using 81% Ph.E, 5% Ph.O, and 14% Ph.P, and compared it with experimental mixtures simulating differences in arrival order through high or low starting concentrations of each strain.

The recreated natural strain assemblage (81% Ph.E, 5% Ph.O, and 14% Ph.P) was the most luminous overall, with an average of 5.0 × 10^7^ ± 3.6 × 10^6^ RLUs, closely followed by the Ph.E-dominated experimental mixture at 4.8 × 10^7^ ± 2.9 × 10^6^ RLUs. Both were significantly more luminous than all other compositions (Supplementary Fig. S5, Supplementary Table S9). Ph.E was the most abundant strain in both cases. Mixtures with low concentrations of Ph.E were consistently dimmer, with the Low Ph.E – High Ph.O – Low Ph.P treatment being the darkest (average = 1.6 × 10^7^ ± 7.4 × 10^5^ RLUs). Although Ph.P was the brightest strain in isolation (Fig. 1B), mixtures in which it was most abundant were not especially bright (Supplementary Fig. S5). Across all mixtures, brightness ranged from 1.3 × 10^7^ to 5.7 × 10^7^ RLUs, overlapping with Ph.P grown in isolation (Fig. 1B, 1.6 × 10^7^–5.5 × 10^7^ RLUs). This range was narrower than that observed across individual strains.

### Priority effects influenced bioluminescence over time

Strain luminescence varied over time, and both monocultures and mixed cultures exhibited distinct temporal dynamics (Fig. 4A and B). Luminescence was not constant but instead followed a double-peaked pattern, with an initial peak occurring between 6 and 9 h and a secondary peak between 13 and 18 h (Supplementary Table S13). Maximum luminescence during the first peak was observed for the Early Ph.E – Early Ph.O – Early Ph.P mixture (7.5 h), the Early Ph.E – Late Ph.O – Early Ph.P mixture (7.6 h), the original composition (7.8 h), and the Early Ph.E – Early Ph.O – Late Ph.P mixture (9.7 h).

**Figure 4:**
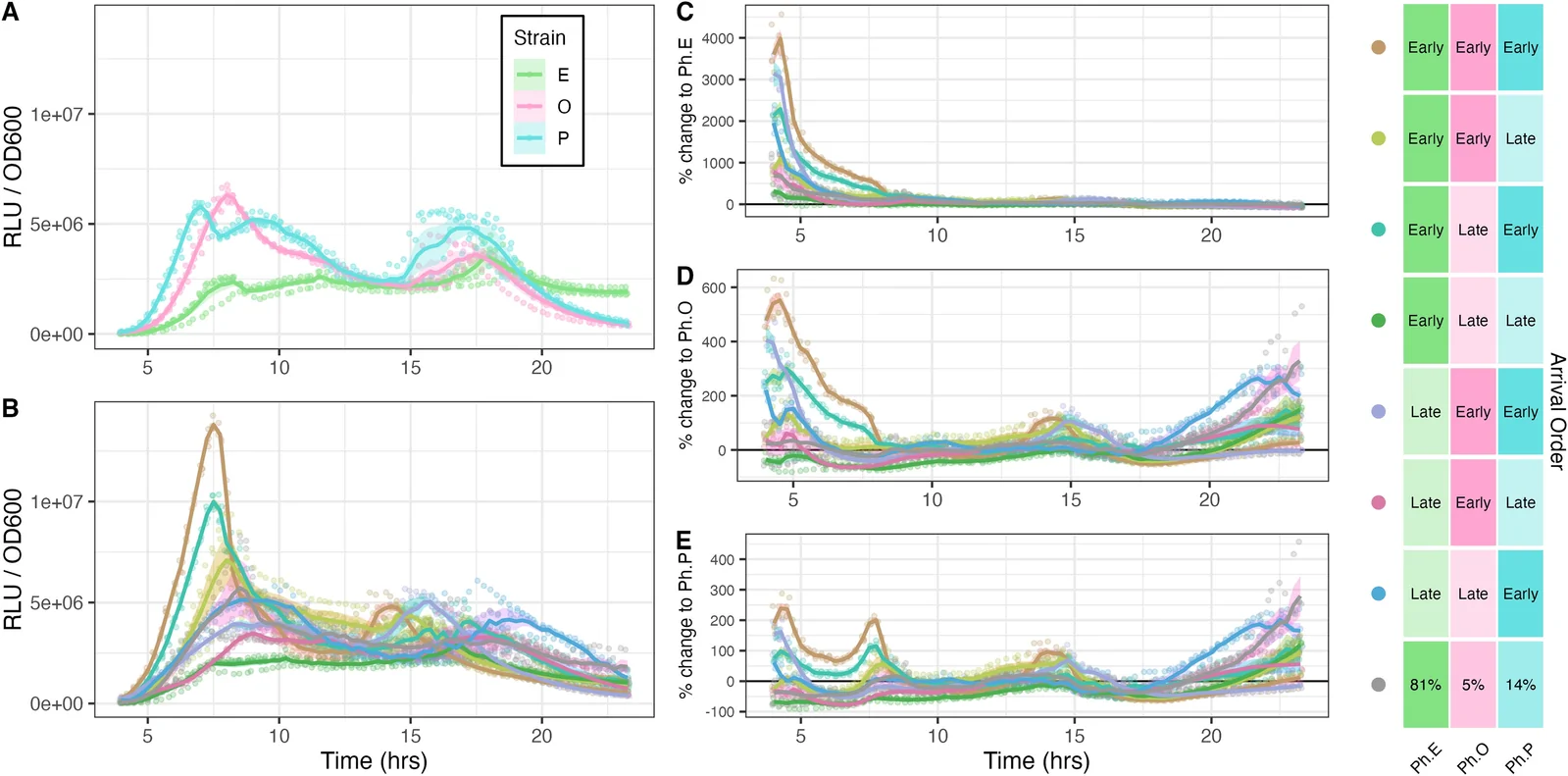
Temporal luminescence of *P. mandapamensis* strains and mixed cultures reveals that light production depends on strain context; luminescence standardized by cell density (RLU/OD_600_) was measured over 24 h for strains Ph.E, Ph.O, and Ph.P grown individually (A) and in mixed cultures (B, *n* = 4); points represent individual measurements, and lines show mean trajectories with shaded ribbons indicating mean ± standard error; percent change in luminescence of each mixture was calculated relative to monoculture references Ph.E (C), Ph.O (D), and Ph.P (E); percent change was calculated at each time point relative to the mean luminescence of the corresponding monoculture reference; values near zero indicate no difference from the reference strain; the tile legend indicates arrival timing for each treatment.

For all remaining mixtures, maximum luminescence occurred during the second peak. Most assemblages in which Ph.E was introduced 4 h later reached their maximum during this later window (Supplementary Table S13). The only exception was the Early Ph.E – Late Ph.O – Late Ph.P mixture, which also peaked during the second window and exhibited the latest peak overall at 17 h.

Strains grown in isolation differed in both the magnitude and timing of peak luminescence (Fig. 4A; Supplementary Fig. S8). The highest light output observed was 6.3 × 10^6^ ± 1.8 × 10^5^ RLUs at ~8 h by Ph.O (Supplementary Fig. S8). However, Ph.P had the highest average luminescence overall (3.1 × 10^6^ ± 5.3 × 10^4^ RLUs). Both Ph.O and Ph.P reached peak luminescence between 8 and 9 h, while Ph.E peaked much later at ~18 h (Fig. 4A; Supplementary Fig. S6). Despite these differences, monocultures did not differ substantially in the stability of light production (Supplementary Fig. S9; Supplementary Table S12).

In mixed cultures, luminescence differed significantly across treatments and was influenced by arrival order (Fig. 4B; Supplementary Figs S6 and S8). The brightest mixture (all *P* < .001), and the highest luminescence observed overall, occurred when all strains were introduced together (1.4 × 10^7^ ± 1.5 × 10^5^ RLUs at 7.5 h). This mixture was brighter than all individual strains until after the first peak in luminescence (Fig. 4C–E). Mixtures in which strains were introduced sequentially varied in brightness and stability, exhibiting reduced brightness and delayed peak timing relative to simultaneously inoculated mixtures (Fig. 4B; Supplementary Figs S6 and S8). The Early Ph.E – Late Ph.O – Late Ph.P mixture, which closely resembled the original composition, had one of the lowest average luminescence values (1.7 × 10^6^ ± 7.5 × 10^4^ RLUs) and a delayed peak at ~17 h, although it maintained a relatively stable light output over time compared to brighter mixtures (Supplementary Fig. S9). Overall, treatments differed in maximum luminescence before 10 h, peak timing, and the stability of light output.

Across all treatments, luminescence could not be predicted from monoculture performance alone. Percent-change analyses relative to Ph.E, Ph.O, and Ph.P monocultures (Fig. 4C–E) showed that many mixtures exceeded monoculture brightness, particularly during the first peak of luminescence. After the second peak (past ~20 h), most mixtures remained brighter than individual strains. However, the magnitude and timing of these effects varied across treatments, and the second peak in mixtures was not consistently higher than monoculture values.

## Discussion

Our results indicate that arrival order can shape the strain composition of *P. mandapamensis* (Figs 2 and 3) and their bioluminescent output (Fig. 4). Strains introduced earlier or at higher initial abundance consistently dominated mixed cultures, demonstrating strong priority effects *in vitro* (Figs 2 and 3). Despite clear differences in luminosity among individual strains (Fig. 1), the brightness of mixed cultures was not predictable from the performance of strains grown in isolation (Fig. 4). In several cases, mixtures were substantially brighter than any individual strain, reaching luminescence levels up to 4000% higher than individual strains (Fig. 4C–E).

Bioluminescence is the defining functional output of the *Siphamia–Photobacterium* symbiosis from which the strains studied here were originally isolated [19, 20]. Measuring luminescent output *in vitro* allowed us to examine how priority effects might influence this symbiotically relevant function. *In vivo*, it would be difficult to control the timing and abundance of symbiont exposure or to measure luminescence dynamics over time. By focusing on strain–strain interactions, our results show that priority effects alone might structure strain composition and influence light output. These findings provide a basis for future *in vivo* experiments aimed at determining how hosts modify or stabilize these interactions. For example, the *S. tubifer* light organ contains hundreds of chambers that may create spatial structure among symbionts [19, 21]. Similar spatial partitioning has been observed in the Hawaiian bobtail squid, where strains of *Vibrio fischeri* rarely co-infect the same crypt [16, 28]. In the *S. tubifer* light organ, too, each chamber houses one strain. As new chambers form, priority effects could independently unfold and collectively shape the distribution of symbionts across the light organ.

Previous work has shown that individual hosts harbor unique strain assemblages with limited sharing across individuals [22]. New strains are not acquired by the host once the initial acquisition period has closed [21, 29]. Our findings suggest that differences in arrival history could be a reason for the high strain variation observed among hosts. For example, the dominance of strain Ph.E over Ph.O and Ph.P observed in one fish may reflect historical contingency. Ph.E may have established in the light organ early rather than outcompeting Ph.O or Ph.P through a trait such as a faster growth rate (Fig. 1). In this system, the precise window of symbiont acquisition has not been fully described, but the light organ becomes receptive to colonization by symbionts at least 7 days after the host enters its pelagic phase [19]. During this time, the host is likely exposed to a diverse environmental pool of potential symbionts before eventually settling onto a *Diadema* urchin alongside other *S. tubifer*. Based on our *in vitro* results, differences in which strains are encountered first could lead to different strain compositions within hosts. Such variability in arrival history may be common in horizontally transmitted symbioses, where symbionts are acquired from the environment [2, 17, 30].

Across all experimental competition conditions, our results were consistent (Figs 2 and 3; Supplementary Figs S2–S4). Strains that arrived early or were introduced at higher initial abundance consistently dominated the final composition (Fig. 2). We conducted parallel experiments manipulating arrival timing and initial inoculum concentration because altering starting abundance has previously been used as a proxy for arrival order in studies of priority effects [26, 27]. In our system, both approaches produced equivalent strain compositions, suggesting that manipulating inoculum abundance can serve as a reliable proxy for arrival timing (Figs 2 and 3). This validation may prove useful for future *in vivo* experiments where precisely controlling strain arrival timing may be difficult. However, these patterns may differ *in vivo*, where hosts could modify competitive interactions and alter strain assembly dynamics.

By measuring the growth rate and bioluminescence of the wild-type strains, we detected phenotypic variation among them (Fig. 1). Ph.E exhibited the highest growth rate but relatively lower luminescence compared with the other strains (Figs 1A and B, and  4A; Supplementary Figs S6–S8), suggesting a potential trade-off between growth and light production. Similar patterns have been observed in other strain combinations of *P. mandapamensis* [22], although evidence for a consistent trade-off between growth and light production remains inconclusive. The strains examined here exhibited measurable differences in growth and light production (Fig. 1). These findings indicate that even small genomic differences among closely related strains can result in detectable phenotypic variation.

Despite these phenotypic differences, strain traits alone did not strongly predict competitive outcomes (Fig. 3). Even when strains were introduced simultaneously at equal densities, no strain consistently excluded the others over the course of the experiment (Figs 2 and 3; Supplementary Fig. S4). Instead, final strain composition was largely determined by arrival history rather than intrinsic differences in growth or luminescence. In other words, which strain arrived first had a greater influence on composition rather than how fast strains grew.

Bioluminescence exhibited a double peak for both isolated strains and mixed assemblages (Fig. 4; Supplementary Fig. S6). Similar patterns have been observed in *Photobacterium kishitanii* and may arise from changes in oxygen availability, cell division dynamics, or luciferase activity [31]. *P. mandapamensis* lacks *LuxI/LuxR*-type quorum-sensing control of luminescence, and the closely related *Photobacterium leiognathi* strain KNH6 shows light production that is not strictly cell-density dependent, instead being strongly influenced by central carbon metabolism and regulators [32, 33]. This density-independent regulation, together with evidence that luciferase activity can report shifts between glycolytic and tricarboxylic acid cycle–based metabolism in *Escherichia coli*, suggests that the biphasic light dynamics we observed might reflect metabolic transitions or metabolite exchanges among strains rather than quorum sensing [34].

While manipulating starting concentration or arrival timing resulted in predictable and reproducible strain compositions (Fig. 2), these treatments produced different luminescence outcomes (Fig. 4). Mixtures reconstructed to match the natural strain composition reported in Gould *et al*. [22] (81% Ph.E, 5% Ph.O, and 14% Ph.P) were the brightest observed when strains were mixed at those proportions and measured immediately (Supplementary Fig. S5). However, when luminescence was measured over time, neither the proportional mixture nor the Early Ph.E – Late Ph.O – Late Ph.P mixture remained the brightest, and both exhibited distinct luminescence trajectories despite their similar compositions (Fig. 4B; Supplementary Figs S6 and S7). Instantaneous luminescence measurements (Supplementary Fig. S5) and measurements taken immediately following the addition of late-arriving strains at 4 h (Supplementary Fig. S7) did not exhibit similar trends either. These differences suggest that strains may modify their environment over time, such that the history of strain interactions influences functional outcomes even when final composition appears similar. In other words, strain assemblages that reach the same strain ratios through different assembly histories produce different functional outputs. This pattern is consistent with previous work where manipulating the timing of species arrival can have stronger effects on function than altering only initial abundance as a proxy for priority effects [35–39].

Bioluminescence measured in mixed cultures further differed from expectations based on individual strain performance (Fig. 4C–E). Several strain compositions produced higher peak luminescence than any individual strain and exhibited distinct temporal changes, indicating that strain interactions can alter the magnitude of bioluminescent output (Supplementary Fig. S8). For example, mixtures dominated by Ph.E produced some of the brightest outputs despite Ph.E producing relatively low luminescence when grown alone (Figs 1 and 4; Supplementary Figs S6–S8). Conversely, Ph.P and Ph.O did not consistently produce the brightest mixtures when abundant (Fig. 4; Supplementary Fig. S8). The brightest outputs occurred when multiple strains were introduced simultaneously, suggesting that early interactions among strains may enhance light production (Supplementary Fig. S8). However, these mixtures also showed less stable luminescence trajectories over time than those assembled through staggered arrival (Supplementary Fig. S9). Because luminescence was normalized to optical density, these differences likely reflect changes in per-cell light production rather than differences in biomass. Although mechanisms remain unclear, shifts in metabolic state, resource availability, or oxygen dynamics may influence light production [31–34]. Such shifts could also reflect a form of metabolic division of labor, in which strains perform complementary roles that reduce individual metabolic burdens while sustaining overall output [40]. Together, these findings show that bioluminescence is an emergent property of strain interactions whose magnitude and stability depend on assembly history.

Our results suggest that priority effects may structure strain composition and shape functional output. Strains exhibited distinct phenotypes and interacted in ways that produced unique patterns of bioluminescence. Bacterial strain composition alone did not fully predict bioluminescent output. Instead, outcomes depended on assembly history. These results will need to be verified *in vivo* to understand the role of priority effects in this symbiosis.

## Supplementary Material

Supplementary_Figure_ycag279

ER_MegaSupplementary_ycag279

Roman_Supplementary_FigureCaptions_Revised_ycag279

## Data Availability

The datasets generated and analyzed during the current study are available in the supplementary materials, and R code can be accessed through https://zenodo.org/records/21125802.
