# Supplementary material for: Bioluminescence may be affected by strain assembly history in a marine symbiont
Source: ISME Commun. 2026 Oct 1;6(1):ycag279. doi: 10.1093/ismeco/ycag279 (PMC13685783; doi:10.1093/ismeco/ycag279)
Supplement: Supplementary file 1 [file roman_supplementary_figurecaptions_revised_ycag279.docx]

**Supplementary**

Figure S1: A box plot showing the growth rates for strains Ph.P, Ph.O, and Ph.E with various fluorescent tags. Strains sharing the same letter are not significantly different from one another (*p* > 0.05). Ph.P did not have any fluorescent tags, so the wild type was chosen. For Ph.O, the mRuby tag was chosen, and for Ph.E the mVenus tag was chosen.

Alternative Text Figure S1: Box plot comparing growth rates of Ph.E, Ph.O, and Ph.P with different fluorescent tags. Growth rate varied among strain and tag combinations. The selected variants were wild-type Ph.P, mRuby-tagged Ph.O, and mVenus-tagged Ph.E.

Figure S2: Initial abundance influenced the composition of pairwise *Photobacterium mandapamensis* strain cultures after 10 hours of growth on solid media. Ph.E is shown in green, Ph.O in pink, and Ph.P in blue. Strain abundance was measured by colony counts. The dashed line indicates the threshold of 10 colonies, below which counts were considered unreliable for calculating CFU/mL. Plates with zero colonies were assigned a value of 1 to reflect very low abundance rather than true absence. The treatment conditions are indicated on the X axis. Upward-pointing triangles denote strains that were introduced at a higher concentration; downward-pointing triangles indicate strains at a lower concentration.

Alternative Text Figure S2: Plot showing abundance of Ph.E, Ph.O, and Ph.P in pairwise strain mixtures after 10 hours under different initial abundance treatments. Strains introduced at higher initial concentrations generally reached higher abundances than strains introduced at lower concentrations.

Figure S3: Initial abundance influenced the composition of mixed *Photobacterium mandapamensis* strain cultures after 10 hours of growth on solid media. Ph.E is shown in green, Ph.O in pink, and Ph.P in blue. Strain abundance was measured by colony counts. The dashed line indicates the threshold of 10 colonies, below which counts were considered unreliable for calculating CFU/mL. Plates with zero colonies were assigned a value of 1 to reflect very low abundance rather than true absence. The treatment conditions are indicated above each panel. Upward-pointing triangles denote strains that were introduced at a higher concentration; downward-pointing triangles indicate strains at a lower concentration.

Alternative Text Figure S3: Multi-panel plot showing abundance of Ph.E, Ph.O, and Ph.P in three-strain mixtures after 10 hours under different initial abundance treatments. Strains introduced at higher initial concentrations generally reached higher abundances relative to strains introduced at lower concentrations.

Figure S4: Initial abundance and arrival order influenced the composition of mixed *Photobacterium mandapamensis* strain cultures after 24 hours of growth. Ph.E is shown in green, Ph.O in pink, and Ph.P in blue. Strain abundance was measured using flow cytometry. Priority effects were simulated by staggering arrival order (*A*) or altering initial strain concentrations as a proxy for arrival order (*B*). The treatment conditions are indicated above each panel. Upward-pointing triangles denote strains that arrived first or were introduced at a higher concentration; downward-pointing triangles indicate strains that arrived later or at a lower concentration. Each point represents the average abundance across biological replicates (n = 4), with black points representing means ± standard error indicated by black bars. Strains sharing the same letter are not significantly different from one another. In both experimental setups, strains introduced first or at higher concentrations maintained higher abundance relative to their competitors. These results show priority effects have a strong role in shaping strain composition.

Alternative Text Figure S4: Multi-panel figure comparing strain abundance after 24 hours when priority effects were done by manipulating arrival order or initial concentration. In both experiments, earlier-arriving strains or strains introduced at higher concentrations generally maintained higher abundances than their competitors.

Figure S5: Strain composition influenced luminescence in mixed *Photobacterium mandapamensis* cultures. Ph.E is shown in green, Ph.O in pink, and Ph.P in blue. Luminescence was measured immediately after mixing standardized overnight cultures from Figure 1B (n = 8). Strain compositions are listed on the x-axis using high or low concentrations. The naturally occurring strain composition was recreated using 81% Ph.E, 5% Ph.O, and 14% Ph.P [22]. Individual data points are shown, with means ± standard error indicated by black bars. Strains sharing the same letter are not significantly different from one another (p > 0.05). Although Ph.P had the highest luminescence when grown alone (Figure 1), mixtures were brightest when Ph.E was present at high concentration.

Alternative Text Figure S5: Plot comparing bioluminescence among three-strain mixtures with different concentrations of Ph.E, Ph.O, and Ph.P. Mixtures containing Ph.E at high concentration produced the most light, despite Ph.P being the brightest when grown alone.

Figure S6: Luminescence and growth dynamics differed among Photobacterium mandapamensis strains grown individually and in mixed culture over 24 hours. Ph.E is shown in green, Ph.O in pink, and Ph.P in blue. Luminescence (relative light units, RLU) and growth (OD600) were measured for strains grown individually (top) and in mixed cultures (bottom). Luminescence values were standardized by cell density (RLU/OD600). Individual data points are shown, with trendlines representing mean luminescence over time (n = 4). Colored points represent luminescence measurements, while grey points represent growth trajectories (OD600) for each treatment.

Alternative Text Figure S6: Multi-panel figure showing bioluminescence and growth over 24 hours for Ph.E, Ph.O, and Ph.P grown individually and in three-strain mixtures with different arrival orders. Bioluminescence and growth trajectories varied among individual strains and mixtures over time.

Figure S7: Luminescence of *Photobacterium mandapamensis* strains and mixed cultures at 4 hours. Luminescence standardized by cell density (RLU/OD600) were measured 4 hours after inoculation, when late strains were introduced. Individual data points are shown with means ± standard error indicated by black bars (n = 4). Strains sharing the same letter are not significantly different from one another (*p* > 0.05). The tile legend indicates arrival timing, where darker tiles represent strains introduced at t = 0 hours (early) and lighter tiles represent strains introduced at t = 4 hours (late).

Alternative Text Figure S7: Plot comparing luminescence standardized by cell density among individual *Photobacterium mandapamensis* strains and three-strain mixtures 4 hours after inoculation. Luminescence differed among strains and mixtures with different arrival histories.

Figure S8: Maximum luminescence of *Photobacterium mandapamensis* strains and mixed cultures within the first 10 hours of growth. Luminescence was standardized by cell density (RLU/OD600), and the 10-hour cutoff was selected to capture the first peak in luminescence observed across treatments. Individual data points are shown with means ± standard error indicated by black bars (n = 4). Strains sharing the same letter are not significantly different from one another (*p* > 0.05). The tile legend indicates arrival timing, where darker tiles represent strains introduced at t = 0 hours (early) and lighter tiles represent strains introduced at t = 4 hours (late).

Alternative Text Figure S8: Plot comparing maximum luminescence standardized by cell density during the first 10 hours among individual *Photobacterium mandapamensis* strains and three-strain mixtures. Maximum luminescence differed among strains and mixtures with different arrival histories.

Figure S9: Stability of light output across *Photobacterium mandapamensis* strains and mixed communities. The coefficient of variation of luminescence standardized by cell density (RLU/OD600) was calculated between 5 and 20 hours of growth. This time window was selected to exclude the early lag phase and late-stage variability, focusing on the period when luminescence production was most consistent. Individual data points are shown with means ± standard error indicated by black bars (n = 4). Strains sharing the same letter are not significantly different from one another (*p* > 0.05). The tile legend indicates arrival timing, where darker tiles represent strains introduced at t = 0 hours (early) and lighter tiles represent strains introduced at t = 4 hours (late).

Alternative Text Figure S9: Plot comparing variation in bioluminescence standardized by cell density between 5 and 20 hours among individual *Photobacterium mandapamensis* strains and three-strain mixtures. The stability of light output differed among strains and mixtures with different arrival histories.
