# Supplementary material for: Bioluminescence may be affected by strain assembly history in a marine symbiont
Source: ISME Commun. 2026 Oct 1;6(1):ycag279. doi: 10.1093/ismeco/ycag279 (PMC13685783; doi:10.1093/ismeco/ycag279)
Supplement: Supplementary file 2 [file supplementary_figure_ycag279.zip › Supplementary_Figure_ycag279/Figure_S1_ycag279.pdf]

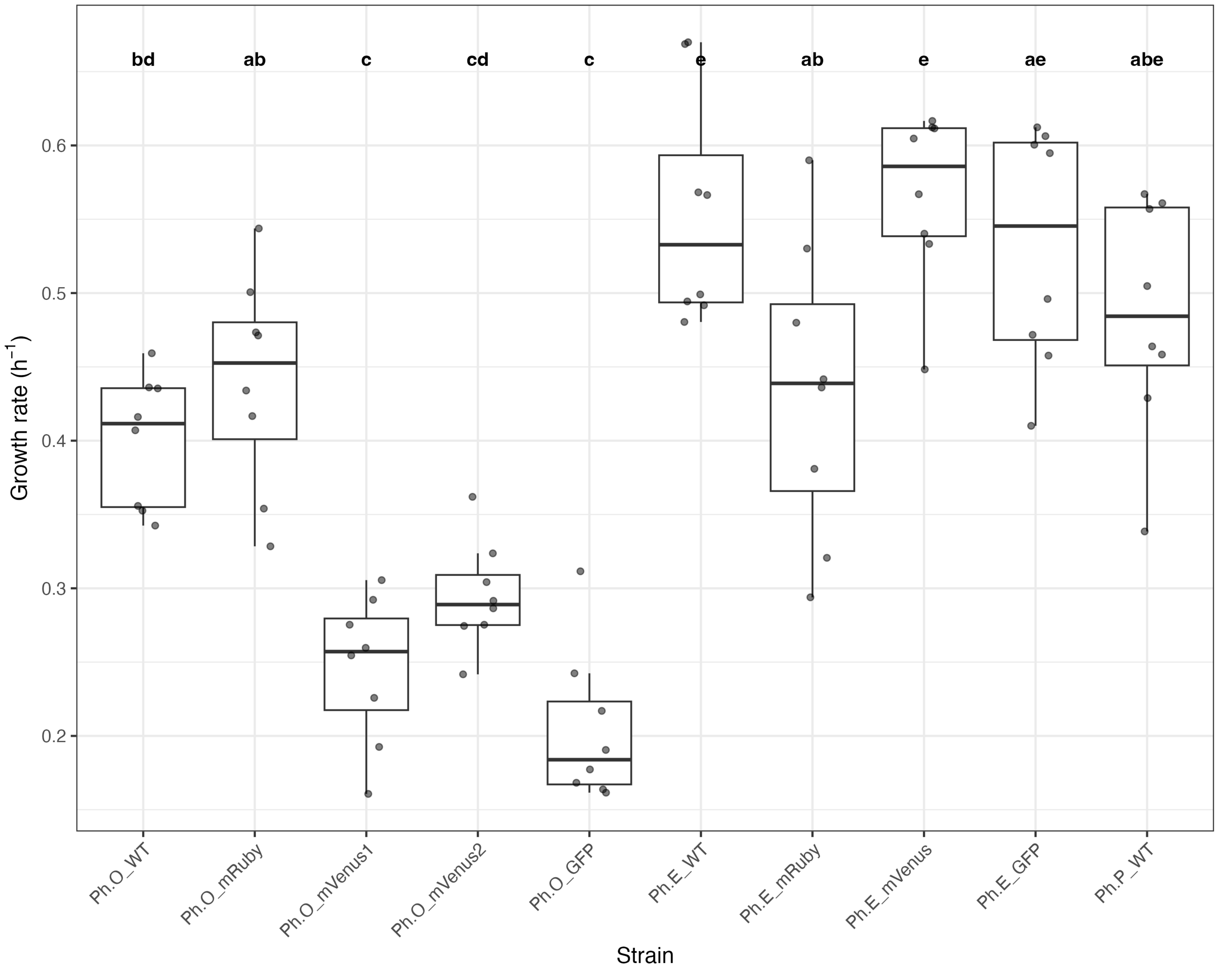

bd
ab
c
cd
c
e
ab
e
ae
abe
0.6
0.5
0.4
0.3
0.2
Growth rate (h^{-1})
Ph.O_WT
Ph.O_mRuby
Ph.O_mVenus1
Ph.O_mVenus2
Ph.O_GFP
Ph.E_WT
Ph.E_mRuby
Ph.E_mVenus
Ph.E_GFP
Ph.P_WT
Strain
