# Supplementary material for: Bioluminescence may be affected by strain assembly history in a marine symbiont
Source: ISME Commun. 2026 Oct 1;6(1):ycag279. doi: 10.1093/ismeco/ycag279 (PMC13685783; doi:10.1093/ismeco/ycag279)
Supplement: Supplementary file 2 [file supplementary_figure_ycag279.zip › Supplementary_Figure_ycag279/Figure_S2_ycag279.pdf]

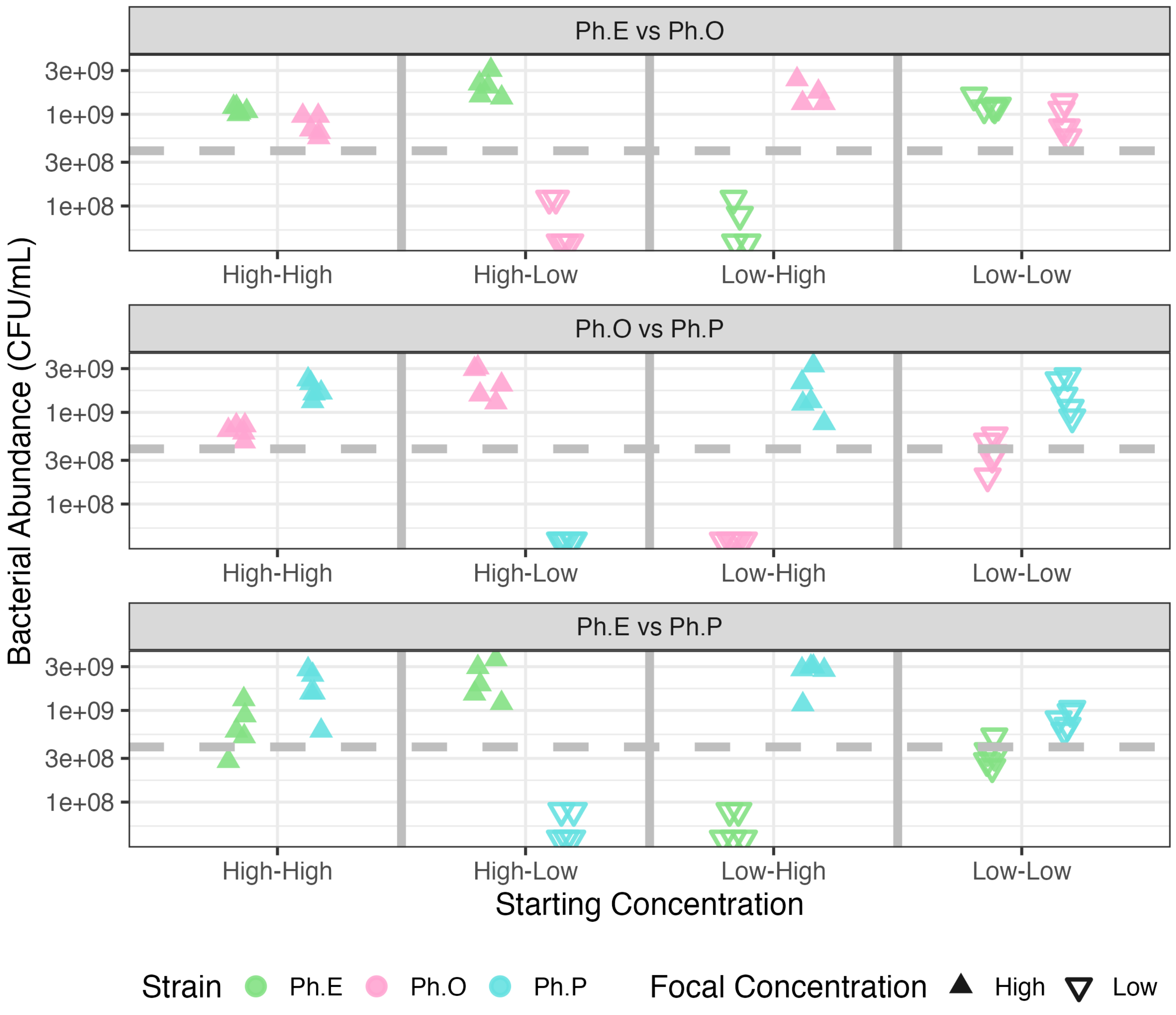

Ph.E vs Ph.O
Ph.O vs Ph.P
Ph.E vs Ph.P
3e+09
1e+09
3e+08
1e+08
High-High
High-Low
Low-High
Low-Low
Bacterial Abundance (CFU/mL)
Starting Concentration
Strain
Ph.E
Ph.O
Ph.P
Focal Concentration
High
Low
