# Supplementary material for: Bioluminescence may be affected by strain assembly history in a marine symbiont
Source: ISME Commun. 2026 Oct 1;6(1):ycag279. doi: 10.1093/ismeco/ycag279 (PMC13685783; doi:10.1093/ismeco/ycag279)
Supplement: Supplementary file 2 [file supplementary_figure_ycag279.zip › Supplementary_Figure_ycag279/Figure_S3_ycag279.pdf]

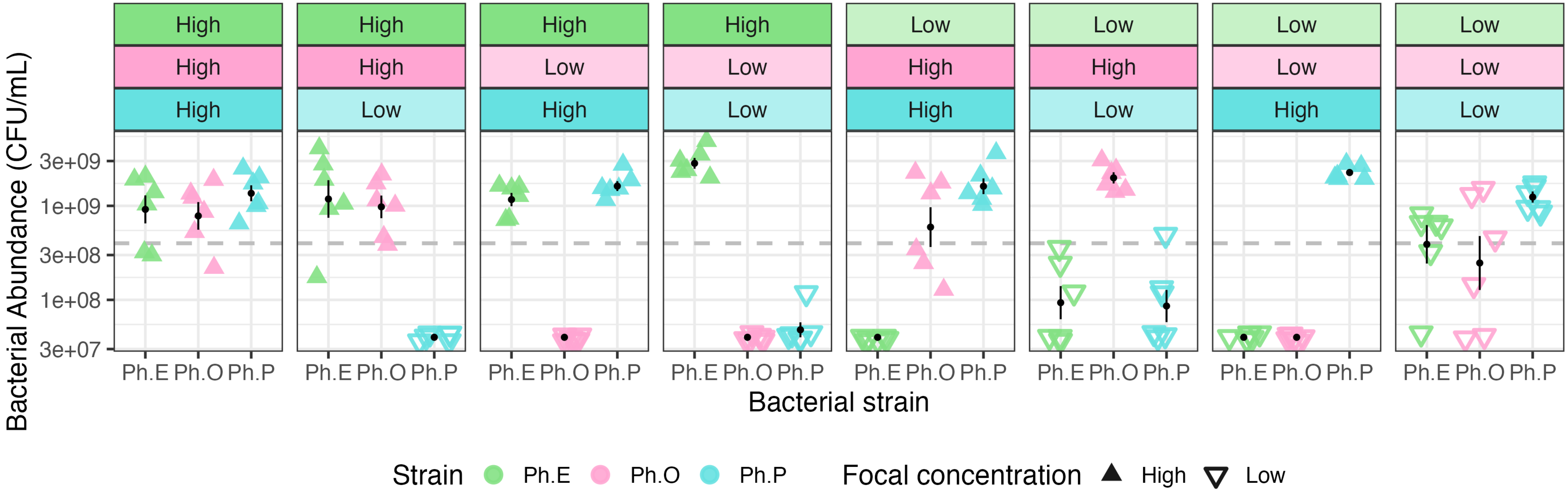

High High High
High High Low
High Low High
High Low Low
Low High High
Low High Low
Low Low High
Low Low Low
Bacterial Abundance (CFU/mL)
3e+09
1e+09
3e+08
1e+08
3e+07
Ph.E Ph.O Ph.P
Bacterial strain
Strain Ph.E Ph.O Ph.P
Focal concentration High Low
