# Supplementary material for: Bioluminescence may be affected by strain assembly history in a marine symbiont
Source: ISME Commun. 2026 Oct 1;6(1):ycag279. doi: 10.1093/ismeco/ycag279 (PMC13685783; doi:10.1093/ismeco/ycag279)
Supplement: Supplementary file 2 [file supplementary_figure_ycag279.zip › Supplementary_Figure_ycag279/Figure_S4_ycag279.pdf]

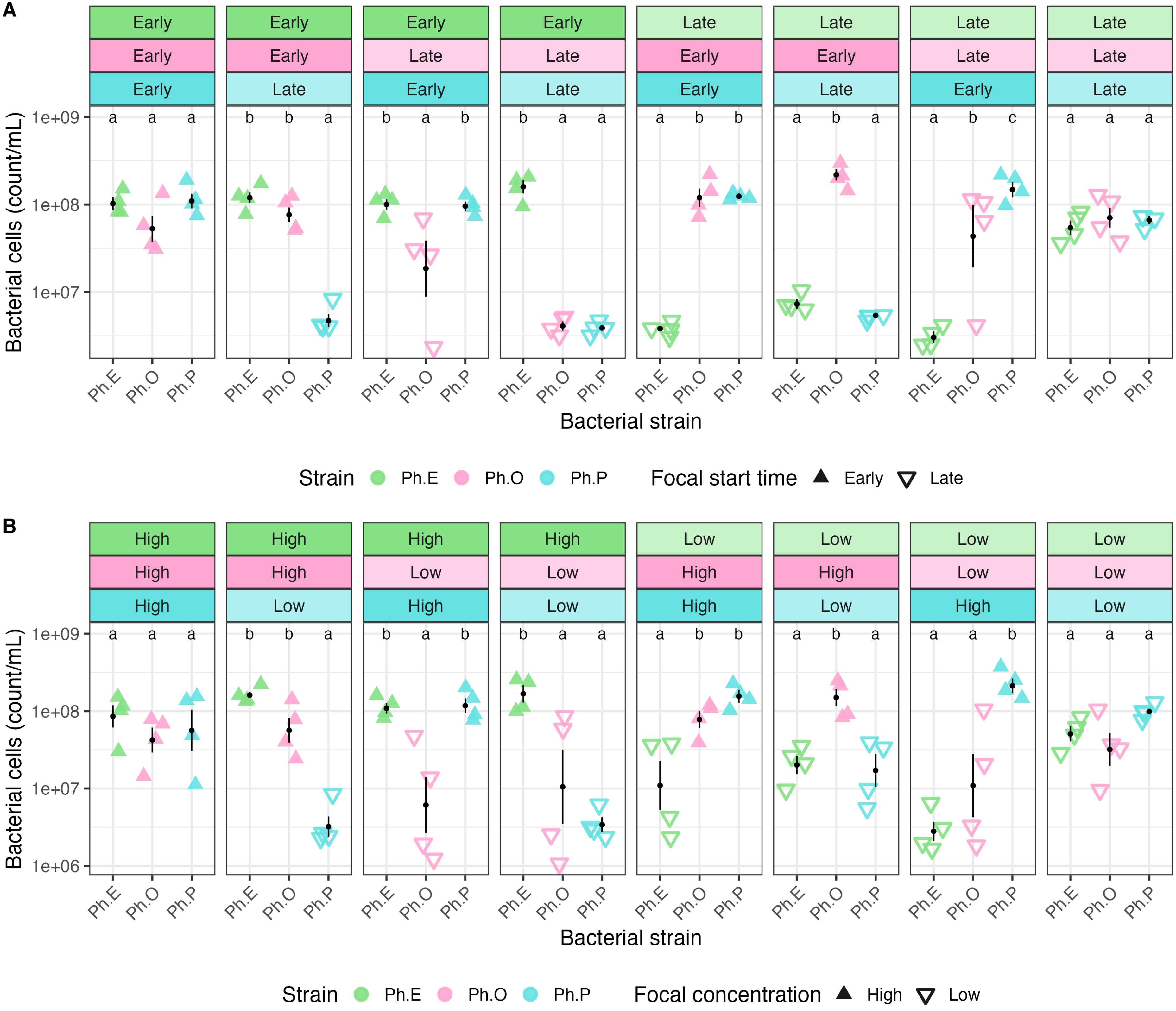
A
Early
Early
Early
Early
Early
Early
Early
Late
Late
Late
Late
Late
Early
Late
Late
Early
Early
Late
Early
Late
Early
Late
Late
Late
a a a
b b a
b a b
b a a
a b b
a b a
a b c
a a a
1e+09
1e+08
1e+07
Bacterial cells (count/mL)
Ph.E Ph.O Ph.P
Bacterial strain
Strain Ph.E Ph.O Ph.P
Focal start time Early Late
B
High
High
High
High
High
High
High
Low
Low
Low
Low
Low
High
High
Low
High
Low
High
Low
High
Low
Low
Low
Low
a a a
b b a
b a b
b a a
a b b
a b a
a a b
a a a
1e+09
1e+08
1e+07
1e+06
Bacterial cells (count/mL)
Ph.E Ph.O Ph.P
Bacterial strain
Strain Ph.E Ph.O Ph.P
Focal concentration High Low
