# Supplementary material for: Bioluminescence may be affected by strain assembly history in a marine symbiont
Source: ISME Commun. 2026 Oct 1;6(1):ycag279. doi: 10.1093/ismeco/ycag279 (PMC13685783; doi:10.1093/ismeco/ycag279)
Supplement: Supplementary file 2 [file supplementary_figure_ycag279.zip › Supplementary_Figure_ycag279/Figure_S5_ycag279.pdf]

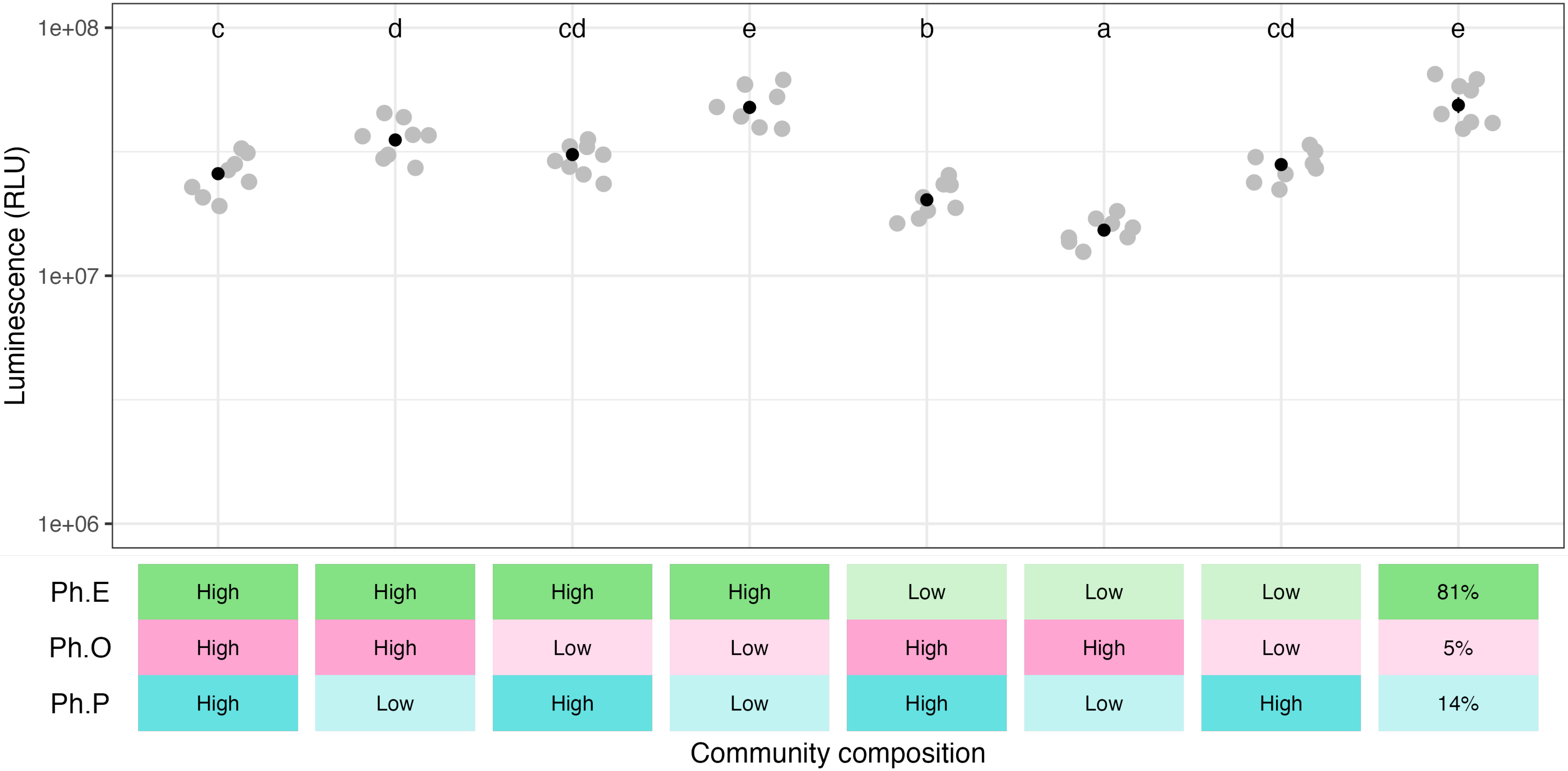

Luminescence (RLU)
1e+08
1e+07
1e+06
c
d
cd
e
b
a
cd
e
Ph.E
Ph.O
Ph.P
High High High High Low Low Low 81%
High High Low Low High High Low 5%
High Low High Low High Low High 14%
Community composition
