# Supplementary material for: Bioluminescence may be affected by strain assembly history in a marine symbiont
Source: ISME Commun. 2026 Oct 1;6(1):ycag279. doi: 10.1093/ismeco/ycag279 (PMC13685783; doi:10.1093/ismeco/ycag279)
Supplement: Supplementary file 2 [file supplementary_figure_ycag279.zip › Supplementary_Figure_ycag279/Figure_S6_ycag279.pdf]

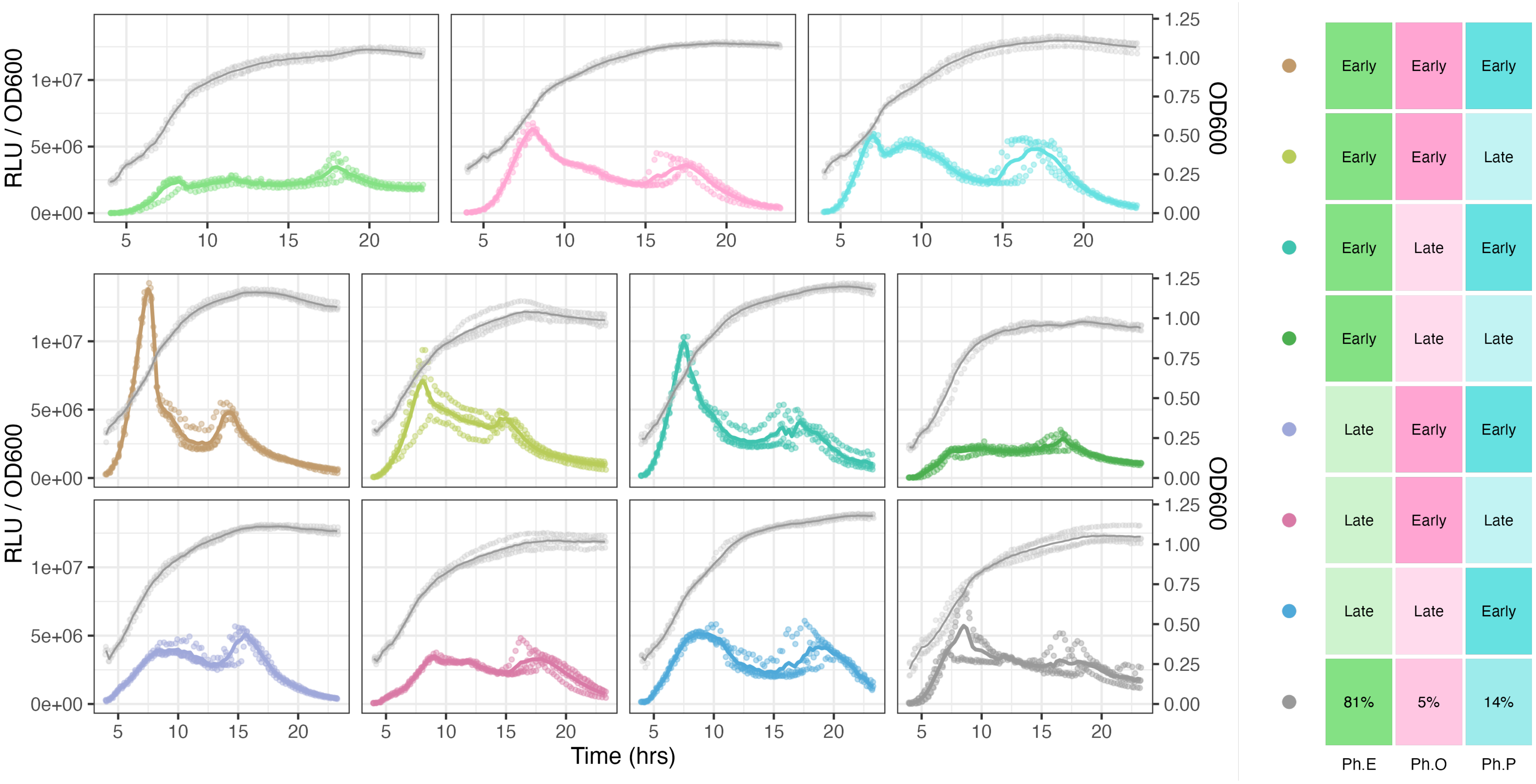

RLU / OD600
OD600
Time (hrs)
1e+07
5e+06
0e+00
0.00
0.25
0.50
0.75
1.00
1.25
5
10
15
20
Early Early Early
Early Early Late
Early Late Early
Early Late Late
Late Early Early
Late Early Late
Late Late Early
81% 5% 14%
Ph.E
Ph.O
Ph.P
