# Supplementary material for: Bioluminescence may be affected by strain assembly history in a marine symbiont
Source: ISME Commun. 2026 Oct 1;6(1):ycag279. doi: 10.1093/ismeco/ycag279 (PMC13685783; doi:10.1093/ismeco/ycag279)
Supplement: Supplementary file 2 [file supplementary_figure_ycag279.zip › Supplementary_Figure_ycag279/Figure_S8_ycag279.pdf]

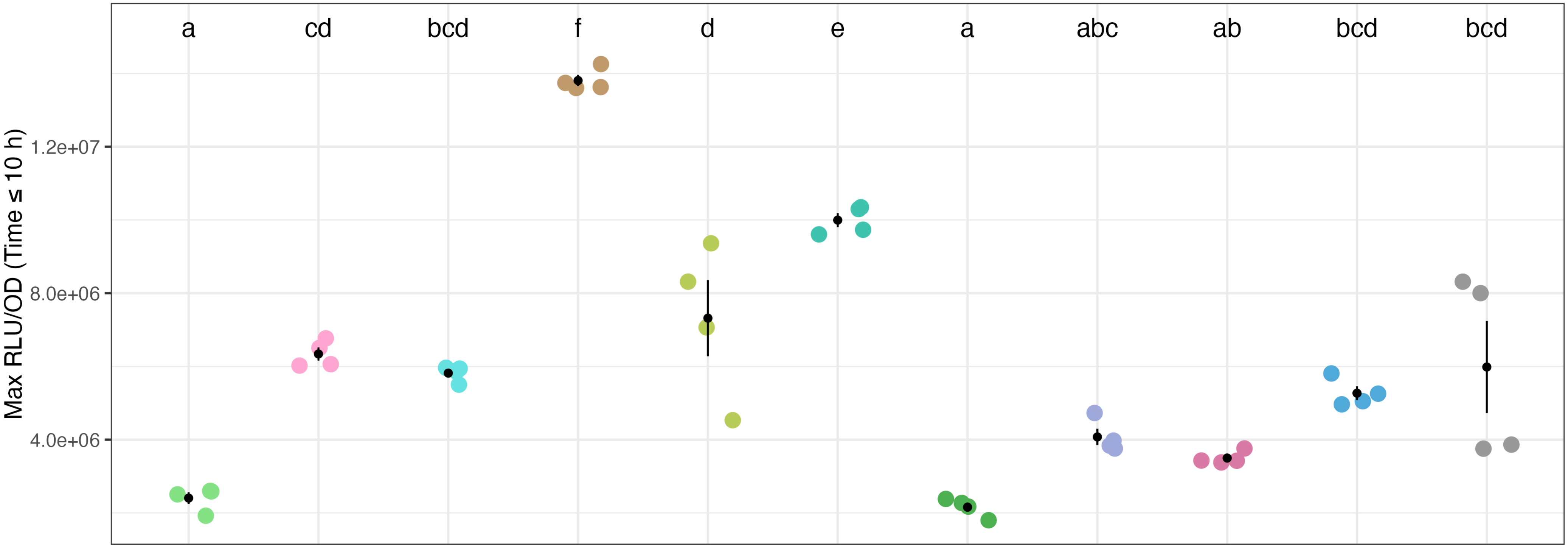

Ph.E	Early	n/a	n/a	Early	Early	Early	Early	Late	Late	Late	81%
Ph.O	n/a	Early	n/a	Early	Early	Late	Late	Early	Early	Late	5%
Ph.P	n/a	n/a	Early	Early	Late	Early	Late	Early	Late	Early	14%
